# The Role of Pharmacists in Travel Medicine in South Africa

**DOI:** 10.3390/pharmacy6030068

**Published:** 2018-07-19

**Authors:** Lee Baker

**Affiliations:** Amayeza Info Services, Johannesburg 1709, South Africa; lee@amayeza-info.co.za

**Keywords:** pharmacists, travel medicine, malaria, malaria prophylaxis, South Africa, schedules

## Abstract

Worldwide, pharmacists, who are the most accessible health-care providers, are playing an ever increasing role in travel medicine, assisting travelers in taking the necessary precautions to ensure safe and healthy travel. This article looks at the situation in South Africa, and how pharmacists are performing these functions within the legal constraints of the Medicines and Related Substances Act 101 of 1965, which prevents pharmacists from prescribing many of the travel vaccines and medications. The scope of practice in community pharmacies increased since the successful down-scheduling of some of the antimalarials, allowing pharmacists to supply the many travelers who frequently travel to neighboring countries. As in many other countries, travel medicine in South Africa is currently thwart with products that are out of stock, and a number of temporary guidelines were put in place to deal with these. Ways to facilitate expanding the role of pharmacists in travel medicine in South Africa need to be further explored.

## 1. Pharmacist Prescribing in South Africa

There are 3370 community pharmacists in South Africa [1], and they potentially have a very important role to play in travel medicine in South Africa, especially with regards to malaria, as there are many malaria-stricken areas within a couple of hours’ travel from people’s homes, which are often visited on weekends. There is also a significant migrant population from neighboring countries that come to South Africa seeking work, and who go home in December (the height of malaria season). Pharmacists are the most accessible health-care professionals, and are, therefore, frequently consulted regarding malaria prophylaxis and other travel health matters. In spite of this, the formal role of pharmacists in travel medicine is in its infancy when compared to some countries such as Canada [2]. This is mainly due to legislature which prevents pharmacists from prescribing or dispensing (without a prescription) any medicine above schedule 2. Medicines in South Africa are scheduled from 0–8, which determines the rules relating to the sale thereof, with schedule 0 (S0) sold in supermarkets, and S3 and up on prescription only. Most travel vaccines are schedule 4 [3].

In addition to the scope of practice of a pharmacist, a pharmacist with the Primary Care Drug Therapy (PCDT) qualification and a Section 22A (15) permit issued by the Director General of Health is permitted to diagnose, treat, and supply medicines following the Primary Health Care Standard Treatment Guidelines and the list of approved medicines, as an authorized prescriber [4].

Section 22A(15) of the Medicines and Related Substances Act (Act 101 of 1965) states that the Director General issues Section 22A(15) permits after consultation with the South African Pharmacy Council (SAPC). Primary Care Drug Therapy (PCDT) permits are issued with a list of conditions and medications that the pharmacist in possession of the permit may prescribe and dispense. This list is in line with the Department of Health’s latest Essential Medicines List. This section reads as follows: “Notwithstanding anything to the contrary contained in this section, the Director General may, after consultation with the Interim Pharmacy Council of South Africa as referred to in Section 2 of the Pharmacy Act, 1974 (Act 53 of 1974), issue a permit to any person or organization performing a health service, authorizing such person or organization to acquire, possess, use, or supply any specified schedule 1, schedule 2, schedule 3, schedule 4, or schedule 5 substance, and such permit shall be subject to such conditions as the Director General may determine [3].” Any application for the scheduling of medicines for this purpose, or for access in terms of Section 22A(15) of the Act should, therefore, use the most recent set of Standard Treatment Guidelines/Essential Medicines List (STG/EML) for Primary Health Care (PHC) issued by the National Department of Health as a starting point, wherever appropriate. The PHC STG/EML is intended to guide the practice of medical practitioners and nurses at PHC facilities in the public sector. Pediatric vaccines against polio, tuberculosis, diphtheria, tetanus, pertussis, hepatitis B, *haemophilus influenzae* type b, measles, pneumococcal, and rotavirus infections are on the Primary Health Care Essential Medicines List, and pharmacists with this Section 22A(15) permit can administer them. The human papillomavirus (HPV) vaccine and the influenza vaccines are also on this list [5]. However, none of the travel vaccines are on this list, and the pharmacist cannot, therefore, prescribe and administer them.

## 2. Pharmacist Activity in Travel Medicine

Currently, 10 pharmacists are members of the South African Society of Travel Medicine (SASTM), and they have all completed the Travel Medicine Course offered once a year by the SASTM, and accredited by the Witwatersrand University. Pharmacists and nurses may only apply to do this course if they have a medical practitioner overseeing them who has either done the course or will do the course with them [6]. This is a very comprehensive course, which equips them with the knowledge they need to be able to offer travel health of the highest standard. This entitles them to apply for a yellow fever license, which allows them to administer these vaccines if they are prescribed by a doctor. Although they cannot prescribe and dispense the necessary vaccines and medicines, they usually work closely with doctors or travel clinics, and play an important role in counseling [3,6]. Most community pharmacists actively counsel travelers on a daily basis, particularly with respect to malaria prophylaxis. Topics that they give advice on, and where possible, products to minimize risks include, traveler’s diarrhea, jetlag, motion sickness, altitude sickness, and prophylaxis of venous thromboembolism [7].

Very few pharmacists currently run their own travel clinics because of the constraints; however, many of the bigger pharmacy groups have started clinics that administer childhood vaccines, and they would be in a good position to open up travel clinics. A few community pharmacists completed the SASTM course and worked under the supervision of a doctor. They are in small rural towns, and they play a very important role.

Two pharmacists, who did the course, worked in a medicine information center, the only privately run one in South Africa. Only one is still employed by the center. Various services are offered, with two of them being a malaria information line and a vaccine information line. Both these services are utilized by health-care professionals, as well as by members of the public. The medicine information center is the Amayeza Info Centre www.amayeza-info.co.za.

Pharmacists have access to a number of resources to assist them with travel health. Those that are members of the SASTM have access to Travax www.travax.nhs.uk and anyone can access the Centers for Disease Control and Prevention (CDC) website for travel health https://wwwnc.cdc.gov/travel, and the World Health Organization (WHO) website for travel and health www.who.int/topics/travel/en/. South African information is available from the National Institute of Communicable Diseases www.nicd.ac.za and the South African National Travel Health Network www.santhnet.co.za.

The current president of the SASTM is a pharmacist, and, in her private capacity, she also sits on the South African Malaria Elimination Committee (SAMEC), which is a committee, made up of experts in the field of malaria, that advises the National Department of Health on malaria. This committee is involved in drawing up the Guidelines for the Treatment of Malaria in South Africa 2018 [8] and the South African Guidelines for the Prevention of Malaria 2017 [9], as well as being instrumental in getting intravenous (IV) artesunate registered in South Africa, and some of the chemoprophylaxis products down-scheduled.

## 3. Antimalarials through Pharmacies

For a medicine to be rescheduled in South Africa, the manufacturer is required to make a submission to the scheduling committee of the Medicines Control Council (MCC), which is now the South African Health Products Regulatory Authority (SAHPRA). In order to make antimalarials more accessible to the public, in the hopes that this would reduce the number of imported malaria cases in South Africa as the country moves toward malaria elimination, the SAMEC approached both the manufacturers and the scheduling committee with a motivation to down-schedule some of the antimalarials. After a number of years of trying to get them down-scheduled to enable a pharmacist to dispense them without a prescription, this was recently achieved. Two years ago, in March 2016, doxycycline [10], and in November 2017, atovaquone-proguanil [11] were cleared to be given out by pharmacists without a prescription. This has enormous benefit for the many travelers who only became aware of the need to obtain antimalarials close to the time of the planned trip, and who did not have sufficient time to arrange for a prescription. In order to ensure that pharmacists are adequately knowledgeable to recommend and dispense these antimalarials, a number of continuing professional development (CPD) talks were given, as well as articles being published in pharmacy and medical journals [12,13].

In the last two years, both South Africa and its neighboring countries have experienced a surge in malaria cases [14]. Namibia experienced four times more cases in 2017 compared to 2015, and incidence rates in Zambia, Mozambique, and Malawi were between 286 and 381 per 1000 people in 2016. Mozambique, which is a popular destination for South Africans, and is also one of the countries from where many of South Africa’s migrant workers come, has between six and eight million cases a year. South Africa’s cases increased from about 5000 cases in 2016 to more than 30,000 cases in 2017 [15]. It is hoped that improving accessibility to antimalarials will result in more travelers taking them, and in a reduction in the number of cases.

## 4. Future Developments

In terms of the Regulations relating to the registration of the Specialities of Pharmacists, Council recognizes Master’s Programs for registration as specialists. There are two specialities currently registrable with Council, i.e., Radio-pharmacy and Clinical Pharmacokinetics [16]. The way forward would be to have travel medicine registered as a speciality. It will then be possible to design a course that will allow pharmacists to prescribe vaccines and medicines appropriate for travel (as the PCDT course only allows them to prescribe for primary care).

## 5. Current Challenges

Travel medicine in general, not specific to pharmacists, saw many challenges in the last year. Many of the travel vaccines and antimalarials were out of stock for months at a time; there is only one manufacturer of pediatric atovaquone-proguanil, and they were out of stock, as was the case with mefloquine, whereas doxycycline cannot be given to children under the age of eight, resulting in no antimalarials available for young children. Vaccine shortages are a worldwide problem; many parts of the world have a yellow-fever vaccine shortage, which South Africa fortunately does not. Both hepatitis A and B vaccines are in short supply, which led to the development of guidelines to deal with these [17,18].

Despite these challenges, travel medicine is alive and well in South Africa, and it is hoped that pharmacists will play an even bigger role in the near future. In a study published earlier this year, clinical outcomes and traveler satisfaction with a pharmacy-based travel clinic was evaluated in Alberta, Canada. Traveler satisfaction was reported as very high with infrequent health issues during travel, and the majority of those who did experience health problems felt that they were adequately prepared to cope with them [2]. These results support an earlier study done in Scotland [19]. Such evidence is important to promote continued expansion of pharmacists' scope in this area, and it is hoped that similar results will be seen in South Africa in the not-too-distant future.
